# Tackling Plant Meiosis: From Model Research to Crop Improvement

**DOI:** 10.3389/fpls.2018.00829

**Published:** 2018-06-19

**Authors:** Christophe Lambing, Stefan Heckmann

**Affiliations:** ^1^Department of Plant Sciences, University of Cambridge, Cambridge, United Kingdom; ^2^Independent Research Group Meiosis, Leibniz Institute of Plant Genetics and Crop Plant Research (IPK) Gatersleben, Seeland, Germany

**Keywords:** meiosis, homologous recombination, crossover, plant breeding, crops, *Arabidopsis thaliana*

## Abstract

Genetic engineering and traditional plant breeding, which harnesses the natural genetic variation that arises during meiosis, will have key roles to improve crop varieties and thus deliver Food Security in the future. Meiosis, a specialized cell division producing haploid gametes to maintain somatic diploidy following their fusion, assures genetic variation by regulated genetic exchange through homologous recombination. However, meiotic recombination events are restricted in their total number and their distribution along chromosomes limiting allelic variations in breeding programs. Thus, modifying the number and distribution of meiotic recombination events has great potential to improve and accelerate plant breeding. In recent years much progress has been made in understanding meiotic progression and recombination in plants. Many genes and factors involved in these processes have been identified primarily in *Arabidopsis thaliana* but also more recently in crops such as Brassica, rice, barley, maize, or wheat. These advances put researchers in the position to translate acquired knowledge to various crops likely improving and accelerating breeding programs. However, although fundamental aspects of meiotic progression and recombination are conserved between species, differences in genome size and organization (due to repetitive DNA content and ploidy level) exist, particularly among plants, that likely account for differences in meiotic progression and recombination patterns found between species. Thus, tools and approaches are needed to better understand differences and similarities in meiotic progression and recombination among plants, to study fundamental aspects of meiosis in a variety of plants including crops and non-model species, and to transfer knowledge into crop species. In this article, we provide an overview of tools and approaches available to study plant meiosis, highlight new techniques, give examples of areas of future research and review distinct aspects of meiosis in non-model species.

## Brief Overview of Meiosis

**FIGURE 1:**
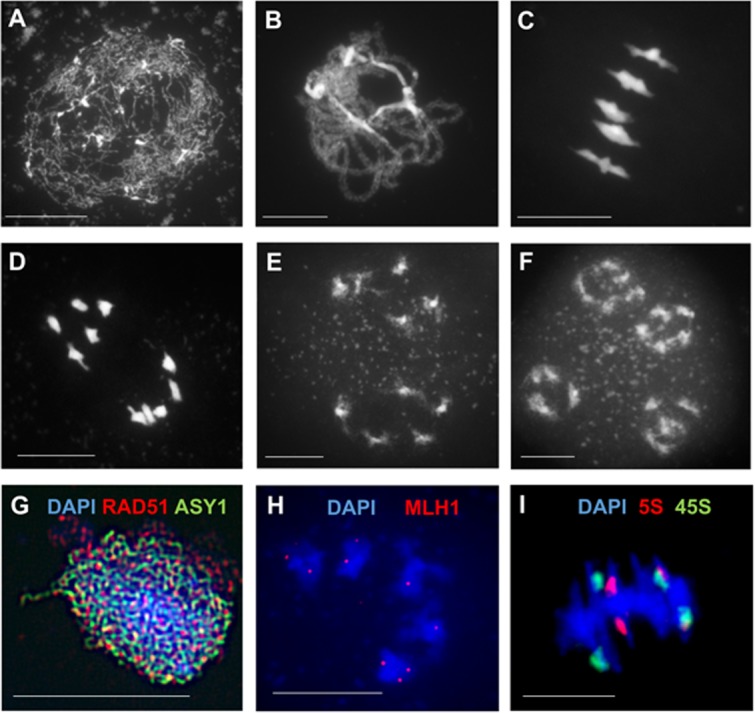
Cytology of male meiosis in *Arabidopsis thaliana.* **(A–F)** Chromosome spreads of Arabidopsis pollen mother cells at different stages during meiosis: **(A)** leptotene, **(B)** pachytene, **(C)** metaphase I, **(D)** anaphase I, **(E)** dyad, **(F)** tetrad. **(G)** Immunolocalization of meiotic-chromosome axis component ASY1 (green) and the recombinase RAD51 (red) at leptotene. **(H)** Immunolocalization of class I CO-marker ZMM protein MLH1 (red) at diakinesis. **(I)** FISH of 45S (green) and 5S (red) rDNA probes at metaphase I discriminates all five pairs of chromosomes forming bivalents. DNA counterstained with DAPI (blue). Scale bar = 10 μM.

Meiosis is a specialized cell division taking place in most sexually reproducing eukaryotic species. It consists of one round of DNA replication followed by two rounds of nuclear division (**Figures 1A–F**). During meiosis, a large number of DNA double-strand breaks (DSBs) are formed and repaired by the homologous recombination (HR) pathway (Osman et al., 2011) (**Figure 1G**). These recombination events are important to bring homologous chromosomes in close juxtaposition and promote crossover (CO) formation (**Figures 1H,I**). A CO is defined as a reciprocal exchange of genetic information between chromosomes. When two polymorphic chromosomes recombine, COs create new combinations of alleles. In addition, COs form physical connections between homologous chromosomes and this ensures correct alignment and segregation of homologous chromosomes on the metaphase plate during meiosis I. At meiosis II sister chromatid cohesion is lost and chromatids segregate to form four haploid recombined gametes. Following this, male and female gametes eventually fuse in the event of fertilization and the diploid state is restored (Mercier et al., 2015).

The meiotic recombination pathway is broadly conserved across plant species although differences exist in the progression of recombination events (Lambing et al., 2017). Meiotic recombination initiates with the formation of DSBs catalyzed by SPO11 and accessory proteins (Robert et al., 2016; Vrielynck et al., 2016). Following DSB formation, SPO11 remains covalently attached to the DSB ends (Neale et al., 2005). DSB ends are then nicked and resected to generate 3′-single-stranded DNA molecules (ssDNAs) (Neale et al., 2005). The recombinases RAD51 and DMC1 bind to the ssDNAs and form nucleoprotein filaments that can anneal to the sister chromatid or to a non-sister homologous chromatid to repair the DSBs (Bishop et al., 1992; Shinohara et al., 1992). During meiosis, a bias in DSB repair toward homologous templates exists (Hong et al., 2013). The majority of these inter-homolog (IH) recombination molecules are eventually displaced by a set of anti-CO proteins and only a subset of these recombination molecules matures in COs (Girard et al., 2014, 2015; Seguela-Arnaud et al., 2015). The fate of recombination molecules is thought to be designated early in prophase I and this seems to be correlated with the accumulation of HEI10 at DSB sites (De Muyt et al., 2014; Lambing et al., 2015). HEI10 is an E3 ligase required for formation of class I COs (Chelysheva et al., 2012; Wang K. et al., 2012). Additional proteins (SHOC1, ZIP4, MSH4/5, MER3, PTD, MLH1/3) are involved in class I CO formation and are collectively named ZMM (Mercier et al., 2015). A second class of COs co-exist independently of ZMM proteins. Class II COs are dependent on structure-specific endonucleases including MUS81 (Berchowitz et al., 2007; Higgins et al., 2008). Class I and II COs differ in their sensitivity to CO interference with the former being sensitive and the latter being insensitive. CO interference is a phenomenon whereby the formation of one CO represses the formation of additional COs in adjacent regions with the strength of the inhibitory effect reducing as the distance increases (Zhang et al., 2014b). The presence of two classes of COs has been observed in Arabidopsis (Higgins et al., 2008) and rice (Zhang P. et al., 2017) and inferred in barley (Phillips et al., 2013) and tomato (Anderson et al., 2014). In Arabidopsis and rice, the proportion of class I COs accounts for ~85–90% of the total COs.

CO distribution appears skewed toward the sub-telomeres in tomato (Demirci et al., 2017), maize (Li et al., 2015), wheat (Choulet et al., 2014), and barley (Phillips et al., 2013). At a finer scale, regions of 1–2 kb with higher recombination rates relative to the genome average have been observed in Arabidopsis, maize and wheat (Choi and Henderson, 2015). Several studies suggest that chromatin features could influence recombination. Repressive epigenetic marks such as DNA methylation and H3K9me2 are enriched over heterochromatin which is repressed for COs in Arabidopsis (Yelina et al., 2012; Yelina et al., 2015). In addition, open chromatin features (H3K4me3 and H2A.Z) are found in CO hotspots (Choi et al., 2013) and RNA-directed DNA methylation at two CO hotspots is sufficient to repress CO formation (Yelina et al., 2015).

Meiotic chromatin is organized in loop-base arrays along a proteinaceous chromosome axis (Kleckner, 2006) and yeast DSBs are formed in the chromatin loops tethered to the axis (Panizza et al., 2011). Components of plant chromosome axes comprise HORMA domain containing proteins (Armstrong et al., 2002; Nonomura et al., 2006), coiled-coil proteins (Wang et al., 2011; Ferdous et al., 2012; Lee et al., 2015) and cohesins (Cai et al., 2003; Lam et al., 2005) and axis mutants show defects in CO formation (Wang et al., 2011; Ferdous et al., 2012; Lee et al., 2015). The composition of the chromosome axis is dynamic and axis re-organization correlates with the progression of DSB repair (Lambing et al., 2015). Genome size and organization differ between plant species (Lambing et al., 2017). For instance, the Arabidopsis genome consists of 20% transposons, which are repetitive DNA elements, while the maize genome consists of 85% transposons. These differences in genome size and organization are associated with changes in chromatin states and epigenetic features and may influence the recombination landscape (Lambing et al., 2017). In addition, findings in Arabidopsis may not be easily transferred to crops. For example, the anti-CO *Atfigl1* Arabidopsis mutant shows increased recombination rates and fertility is unaffected (Girard et al., 2015), while *Osfignl1* rice is infertile (Zhang P. et al., 2017). Therefore, new tools, techniques and approaches are needed to facilitate the investigation of underlying mechanisms and factors responsible for differences between model and crop meiosis, in order to ultimately translate our knowledge into crop breeding programs.

## Imaging Approaches

### Super-Resolution Microscopy

The resolution of fluorescence microscopy is limited to ~200 nm due to the diffraction limit of light, while EM can resolve cellular structures up to ~1 nm revealing ultrastructural meiotic chromosome features in various plants (e.g., Albini and Jones, 1987; Albini, 1994; Anderson et al., 2014). However, fluorescence microscopy enables identification and co-localization of labeled cellular structures and molecules. Super-resolution fluorescence microscopy techniques such as SIM (Structured Illumination Microscopy), PALM (Photoactivated Localization Microscopy) or STORM (Stochastic Optical Reconstruction Microscopy) allow analysis of labeled cellular structures and molecules beyond the diffraction limit of light (“subdiffraction” imaging) in plants (Schubert, 2017). Plant cell imaging is challenging when compared to animal tissues due to high levels of autofluorescence and varying tissue refractive indexes leading to light scattering and spherical aberrations (Komis et al., 2015). Tissue-clearing techniques (Kurihara et al., 2015; Musielak et al., 2016; Nagaki et al., 2017) and substances which shift refraction indexes (Littlejohn et al., 2014) may enable “subdiffraction” imaging in intact plant tissues to study meiosis. Currently meiotic chromosome spreads enable high-resolution imaging in various plant species giving new insights into axis, synaptonemal complex (SC) and CO formation as well as meiotic chromosome organization and segregation (Colas et al., 2017; Schubert, 2017). High-resolution microscopic approaches, including single molecule counting and localization by PALM or STORM implemented for non-meiotic plant cells (Schubert and Weisshart, 2015), will likely ensure further insights into meiotic processes in the future.

### Live Cell Imaging

Most of our knowledge of plant meiotic progression is based on reconstructions made from fixed materials (Sanchez-Moran and Armstrong, 2014). Meiotic live cell imaging could be an instrumental tool to follow meiotic chromosome and recombination dynamics *in planta* improving our understanding of the spatiotemporal progression of meiotic events. It could, for instance, enable a study of the interplay between axis, SC and HR dynamics or lead to a better understanding of spatiotemporal asymmetric meiotic progression in cereals resulting in CO-heterogeneity (Higgins et al., 2012). However, reports on meiotic live cell imaging are limited. Live cell imaging of isolated and cultured maize meiocytes (Yu et al., 1997, 1999; Nannas et al., 2016) deciphered the dynamics and duration of meiosis I and II chromosome segregation and revealed mechanisms correcting off-centered metaphase spindles. Meiocytes were also analyzed within intact anthers of maize during prophase I (Sheehan and Pawlowski, 2009) and within intact anthers and gynoecia of *Arabidopsis thaliana* (Ingouff et al., 2017). In maize, actin- and tubulin-dependent prophase I chromosome movements are rapid and complex including general chromatin rotations and movements of individual chromosome segments (Sheehan and Pawlowski, 2009). In Arabidopsis, live imaging based on fluorescent protein (FP)-tagged proteins revealed the dynamics of DNA methylation before, during and after meiosis (Ingouff et al., 2017). Although an in-depth analysis of male and female meiotic progression was not performed, highly dynamic chromatin movements during male meiosis were described, suggesting similar prophase Ichromosome movements as in maize. Whether similar prophase I chromatin movements, chromosome segregation dynamics or spindle correction mechanisms occur in other plant species; whether chromosome number or genome size/organization have an impact, and how these processes are interrelated with meiotic progression needs to be established.

**FIGURE 2:**
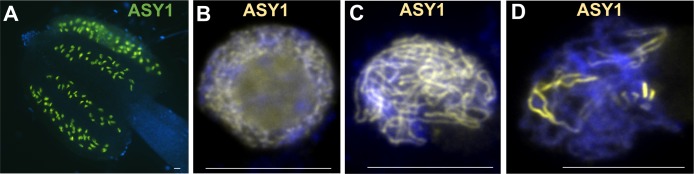
*Arabidopsis thaliana* pollen mother cells expressing FP-TAGged ASY1. **(A)** Squashed anther of *A. thaliana* expressing axis-component ASY1-eGFP (green). **(B–D)** Chromosome spreads of Arabidopsis pollen mother cells expressing ASY1-eYFP (yellow). **(A–D)** No antibody used for detection of ASY1-eYFP. DNA counterstained with DAPI (blue). Scale bar = 10 μM.

In addition to visualizing meiotic proteins based on plants expressing FP-tagged proteins during meiosis (**Figure 2**), the development of CRISPR-imaging (Dreissig et al., 2017b) may enable simultaneous visualization of certain chromosome regions and their dynamics. Tracing single molecule dynamics by CRISPR-PALM in non-plant species (Cho et al., 2016; Khan et al., 2017) as well as live cell SIM imaging and single particle PALM tracking in living plants (Schubert, 2017; Komis et al., 2018) were reported in non-meiotic tissues/cells. Such advanced high-resolution live microscopic imaging applications are challenging for the study of plant meiosis due to the depth of tissue where meiotic cells are embedded, high levels of autofluorescence, light scattering and spherical aberrations. To overcome these plant-specific imaging challenges, the application of multiphoton excitation microscopy (Sheehan and Pawlowski, 2009), two photon excitation microscopy or light sheet fluorescence microscopy may enable meiotic live cell imaging, although not at high-resolution.

## Proteomic Approaches

### Meiotic Proteomes

Most plant meiotic genes were identified through mutant and genetic suppressor screens or based on sequence conservation with other species. An alternative approach is direct candidate identification by “omics” approaches. To identify proteins present during meiosis, proteomics studies were initially performed using two-dimensional electrophoresis and subsequent “spot” identification by mass spectrometry in various plants (e.g., (Kerim et al., 2003; Sánchez-Morán et al., 2005; Imin et al., 2006; Phillips et al., 2008). Proteomes from flower buds, anthers or isolated meiocytes from Arabidopsis (Lu et al., 2016), tobacco (Ischebeck et al., 2014), *B. oleracea* (Osman et al., 2018), tomato (Chaturvedi et al., 2013), rice (Collado-Romero et al., 2014; Ye et al., 2015), or maize (Wang D. et al., 2012; Zhang et al., 2014a) consist of hundreds or thousands of proteins functionally enriched e.g., for (i) mRNA transcription, stability, and processing, (ii) protein synthesis, translation and splicing and (iii) ubiquitin-proteasome system (UPS) function. While there is evidence implicating transcriptional processes (Nan et al., 2011; Zhang et al., 2015) and UPS function (see section “The Ubiquitin-Proteasome System”) in plant meiosis, any direct role of spliceosome or ribosomal proteins in meiotic recombination remains elusive. However, (alternative) splicing is a likely regulatory mechanism during meiosis (Cavallari et al., 2018; Walker et al., 2018).

Meiotic proteome complexity was reduced based on: comparative proteomics combined with transcriptomics in *A. thaliana* (Lu et al., 2016); ASY1 affinity proteomics in *B. oleracea* (Osman et al., 2018); proteomic approaches focusing on the identification of posttranslational protein modifications (PTMs) in rice (Ye et al., 2015; Li et al., 2018). Surprisingly, a comparison of available Arabidopsis flower bud proteomes suggests that protein detection was not saturated (Lu et al., 2016). In addition, proteomes from rice anthers and isolated rice meiocytes identified 6831 and 1316 proteins, respectively (Collado-Romero et al., 2014; Ye et al., 2015). However, only 10 of at least 28 known rice meiotic genes (Luo et al., 2014) were identified, suggesting that even these extensive data sets do not represent the whole meiotic proteome.

### Posttranslational Protein Modifications

In non-plant species SC, axis and HR protein dynamics are regulated via PTMs, such as Ubiquitination and SUMOylation (small proteins conjugated to other proteins regulating target stability and localization or their interaction with further proteins) or phosphorylation, coordinately interlinking meiotic chromosome remodeling and HR spatiotemporally during meiosis I (Carballo et al., 2008; Fukuda et al., 2012; Ahuja et al., 2017; Rao et al., 2017). Despite strong evidence for the essential role of PTMs for proper axis, SC and CO formation in budding yeast and mammals, the role of PTMs of corresponding plant homologs are unknown. However, there is growing evidence that in plants too, PTMs of meiotic proteins are essential for meiosis.

#### Phosphorylation

**FIGURE 3:**
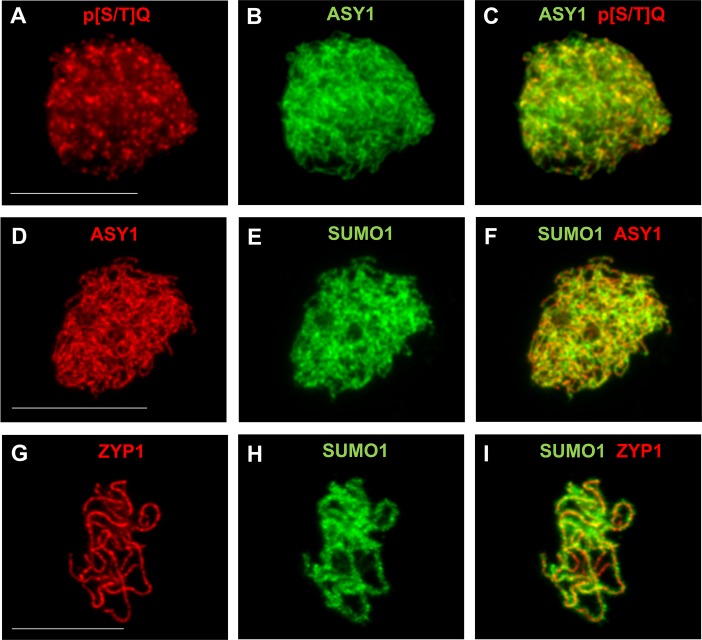
Immunodetection of PTMs on male meiotic *A. thaliana* chromosomes. Immunostaining of ASY1 (green, dilution 1/1000) and phospho-(Ser/Thr) ATM/ATR substrate (p[S/T]Q) (red, Cell Signaling Technology, 2851, dilution 1/2500) at leptotene **(A–C)**, of ASY1 (red, dilution 1/1000) and SUMO1 (green, abcam, ab5316, dilution 1/1000) at leptotene **(D–F)**, and of ZYP1 (red, 1/500) and SUMO1 (green, abcam, ab5316, dilution 1/1000) at pachytene **(G–I)**. Scale bar = 10 μM.

In non-plant species meiotic chromosome axis proteins undergo phosphorylation critical for their function (Brar et al., 2006; Carballo et al., 2008; Fukuda et al., 2012), e.g., budding yeast Hop1 T318-phosphorylation promotes Hop1-dependent IH bias (Carballo et al., 2008) and S298-phosphorylation promotes stable interaction of Hop1 and Mek1 on chromosomes (Penedos et al., 2015). ASY1 (Hop1) affinity proteomics in *B. oleracea* revealed multiple phosphorylated residues in BoASY1 and BoASY3 (Osman et al., 2018) and OsPAIR2 (homolog of BoASY1) is phosphorylated in rice (Ye et al., 2015). Phosphorylation of BoASY1 at T294 and the flanking residue S300 may functionally correspond to Hop1 T318 and the flanking residue S298 (Osman et al., 2018). In rice anthers phosphoproteomics more than 400 of 3203 identified phosphoproteins are meiotically expressed, including 32 known meiotic genes (Ye et al., 2015). A screen for somatic ATM/ATR (serine/threonine protein kinases triggering the DNA damage response) targets in Arabidopsis identified up- and down-regulated phosphorylation of 108 and 32 candidates, respectively, including various proteins with a role in meiotic DNA damage response (Roitinger et al., 2015). In pollen mother cells, immunolocalization of proteins with phosphorylated [S/T]Q residues, substrate of ATM and ATR kinases, revealed numerous foci associated with the chromosome axis (**Figures 3A–C**). Whether identified phosphorylated residues in meiotic candidate genes in rice and Arabidopsis play a role in meiosis is unclear.

#### SUMOylation

SUMOylation is a reversible PTM involved in meiotic chromosome axes remodeling, SC formation and HR in budding yeast and nematodes (Zhang et al., 2014c; Nottke et al., 2017). Loss of Arabidopsis SUMO E3 ligase MMS21 results in meiotic chromosome mis-segregation and fragmentation (Liu et al., 2014). Eight SUMO genes (SUMO1-8) are found in Arabidopsis, but only SUMO1/2/3/5 are expressed (Hammoudi et al., 2016). SUMO1/2 are closely related, redundant for plant viability and highly expressed. Immunolocalization of AtSUMO1 on meiotic chromosomes shows abundant signal on chromatin and the chromosome axis (**Figures 3D–I**). In contrast, SUMO3/5 are more divergent and weakly expressed. A SUMO3 mutant shows no obvious plant development phenotype while data on SUMO5 is limited. However, functional data on meiosis are lacking for all expressed SUMOs except SUMO1 which is present on meiotic chromosomes (**Figures 3D–I**). Advances in MS-based detection of SUMO targets (Rytz et al., 2016) and SUMO pathway mutant studies during meiosis should shed further light on whether SUMOylation plays a key role in meiosis in plants.

#### The Ubiquitin-Proteasome System

In various organisms the UPS is involved in SC and CO formation (Ahuja et al., 2017; Rao et al., 2017). In rice and Arabidopsis, a role for the UPS in meiosis was demonstrated (Yang and McLoud, 2012; He et al., 2016; Zhang F. et al., 2017). In rice, two F-box proteins, MOF and ZYGO1, interact with the rice SKP1-like Protein1 (OSK1), probably as components of the SKP1-CUL1-F-box (SCF) E3 ubiquitin ligase, and are essential for meiosis. MOF regulates male meiotic progression and DSB end-processing and repair (He et al., 2016), whereas ZYGO1 mediates bouquet formation promoting SC and CO formation in both male and female meiosis (Zhang F. et al., 2017). In Arabidopsis, SKP1-like (ASK1) protein (a subunit of the SCF E3 ubiquitin ligase complex) is critical for homologous chromosome pairing, synapsis and nuclear organization during meiosis (Yang and McLoud, 2012) and putative ASK1-substrates include UPS candidates (Lu et al., 2016). Affinity proteomics of the meiotic chromosome axis in *B. oleracea* also identified UPS candidates (Osman et al., 2018).

#### Additional PTMs

NEDD8, another small protein, is involved in Neddylation that is critical for SC and CO formation in *A. thaliana* (Jahns et al., 2014). Mutation in AXR1, the E3-conjugating NEDD8 ligase, results in a reduced number of bivalents and synapsis defects. The reduced number of bivalents is not due to a general CO decrease, rather due to altered class I CO localization and crossover interference resulting in loss of the obligatory CO. In *arx1 zmm* double mutants barely any CO formation occurs indicating that in *axr1*, MUS81-dependent class II CO are probably abolished. Whether further components of the Neddylation system are critical for meiosis and which meiotic proteins undergo Neddylation needs to be established.

Proteomics from rice anthers identified 357 acetylated proteins including eight rice homologs of known meiotic genes (Li et al., 2018). A positive correlation of simultaneous acetylation and phosphorylation of candidates functionally enriched for ribosome assembly, protein translation, UPS, and RNA degradation was found, further linking these processes to plant meiosis (see section “Meiotic Transcriptome”). Acetylation of histones was abundant, various histone acetyltransferases and deacetylases were detected in rice meiotic transcriptomes (Zhang et al., 2015) and GCN5-related histone N-acetyltransferase alters meiotic recombination in Arabidopsis (Perrella et al., 2010), suggesting a link between histone acetylation and meiosis. Interestingly, in rice H3K9 hyperacetylation correlates with meiotic arrest in *mel1* (Liu and Nonomura, 2016), H3K9 acetylation affects yeast recombination hotspots (Yamada et al., 2013) and H4K12/H4K16 acetylation impacts meiotic chromosome segregation in human and mouse (van den Berg et al., 2011; Ma and Schultz, 2013). All of these sites were acetylated in rice (Li et al., 2018).

## Genomic and Transcriptomic Approaches

### Chromatin Immunoprecipitation of Recombination Proteins

Chromatin immunoprecipitation sequencing (ChIP-seq) of recombination proteins consists of precipitating DNA molecules found in complex with proteins. DNA molecules are then detected using Next-Generation sequencing. In plants, the first genome-wide maps of DSBs were generated in maize using a RAD51 antibody (He et al., 2017) and in Arabidopsis using an epitope-tagged SPO11-1-MYC (Choi et al., 2018). DSB hotspots were located in repetitive and gene regions in both species. Similar to yeast (Pan et al., 2011) and mice (Lange et al., 2016), maize and Arabidopsis DSBs are mostly located in nucleosome depleted regions and in regions of low DNA methylation (He et al., 2017). Genome-wide correlation between DSBs and COs is low while a positive correlation between DSBs located in genic regions and COs was found (He et al., 2017), suggesting that DSB formation is not repressed over repetitive regions but recombination outcome differs depending on local features. Interestingly, removal of the heterochromatin silencing marks H3K9me2 and non-CG methylation in Arabidopsis resulted in an increase in DSBs and COs over the pericentromeres (Underwood et al., 2018). Understanding how DSBs are repaired and acquire a CO fate is essential as it could facilitate the manipulation of CO rate over genes of interest.

### Mapping Crossovers

Despite the formation of a large number of meiotic recombination events only a subset of them forms a CO. The remaining recombination molecules are resolved as NCOs (Mercier et al., 2015). NCOs can be accompanied by gene conversions (GCs) which consist of non-reciprocal exchanges of genetic information causing a non-Mendelian 3:1 segregation ratio of alleles (Sun et al., 2012). Several techniques exist to measure CO rate (**Table 1**). For example, transgenic Arabidopsis lines with genetically linked genes expressing FPs either in pollen (Berchowitz and Copenhaver, 2008) or seeds (Melamed-Bessudo et al., 2005) can be used to measure recombination, based on the segregation ratio of the FP-coding genes, in male meiosis and male/female meiosis, respectively. This technique was also adapted to measure GCs and revealed that GC rate is low and estimated at 3.5 × 10^-4^ per locus per meiosis and that the majority of these GCs are associated with a CO, while only a few GCs are associated with a NCO in Arabidopsis (Sun et al., 2012). Unfortunately, the generation of FTLs in crops would be laborious, expensive and time-consuming. As an alternative approach single pollen genotyping was developed in barley (Dreissig et al., 2015). The method consists of isolating individual haploid pollen nuclei from F1 hybrids by utilizing fluorescence activated cell sorting (FACS) followed by whole-genome amplification and subsequent multi-locus KASP-genotyping or single-cell genome sequencing (Dreissig et al., 2015; Dreissig et al., 2017a). This technique has the advantage of analyzing the DNA content from gametes before fertilization so that measurement of CO rate is not affected by segregation distortion.

**Table 1:** Comparison between the different methods to detect COs.

Techniques	Principle	Resolution	Species	Reference
Chromosome spreading combined with FISH and immunolocalization of class I CO marker	Detection of chiasmata based on bivalent morphology and detection of class I COs based on immunolocalization of MLH1/HEI10 in Arabidopsis and MLH3 in Barley	Chromosome scale	Bivalent morphology and FISH: variety of plant species; Immunolocalization of class I CO marker: Arabidopsis, barley, brassica, tomato, rice, wheat	e.g., Arabidopsis (Chelysheva et al., 2012), barley (Higgins et al., 2012)
Fluorescent transgenic lines (FTLs)	Measurement of CO rate in pollen (male) or seeds (male/female) based on the segregation ratio of genetically linked genes expressing fluorescent proteins. Measurement of CO interference	Mbs	Arabidopsis	Pollen (Berchowitz and Copenhaver, 2008), seeds (Melamed-Bessudo et al., 2005)
Genotyping-by-sequencing and molecular markers in segregating F2 populations	Measurement of CO rate in F2 population derived from F1 hybrid based on polymorphisms (primarily SNPs) of parental genomes through low-coverage genome sequencing or selected molecular markers. Genome-wide CO distribution and CO interference can be measured. Molecular markers can also be used to study CO rate at a specific region of the chromosome.	~1 kb depending on SNP density	Arabidopsis, tomato, rice, wheat, and maize	Arabidopsis (Yelina et al., 2015), tomato (Demirci et al., 2017), rice (Si et al., 2015), wheat (Saintenac et al., 2009), maize (Yao and Schnable, 2005)
Microspore (tetrad or pollen nuclei) genotyping or sequencing	Measurement of CO rate in individual microspores (tetrad or pollen nuclei; male meiosis) from F1 hybrid based on polymorphisms (primarily SNPs) of parental genomes through low-coverage genome sequencing or KASP-genotyping. Genome-wide CO distribution and CO interference can also be measured. Detection of gene conversion events if individual nuclei of a tetrad are sequenced. Can differentiate between COs and segregation distortion	~1 kb depending on SNP density (sequencing). The resolution of COs detected using KASP-genotyping depends on the number of markers used	Barley (pollen: genotyping, sequencing), maize (tetrad: sequencing), Arabidopsis (tetrad: sequencing)	Barley (Dreissig et al., 2015, 2017a), Maize (Li et al., 2015), Arabidopsis (Lu et al., 2012)
Pollen typing	Measurement of CO rate in pollen nuclei (male meiosis) from F1 hybrid at specific loci (hot spots) based on polymorphisms (primarily SNPs) of parental genomes through allele-specific PCRs	<1 kb depending on SNP density	Arabidopsis	Yelina et al., 2012, 2015; Choi et al., 2016

Another technique called genotyping-by-sequencing consists of low-coverage sequencing of the genomes of a large F2 population derived from F1 hybrids providing a genome-wide crossover distribution (Si et al., 2015; Yelina et al., 2015). The position of the COs is inferred by detecting changes in single nucleotide polymorphisms (SNP) positions in the F2 population. However, this technique is expensive, the resolution of CO sites is low (>1 kb) and the number of individuals analyzed is limited (Yelina et al., 2015). Nevertheless, this technique revealed that CO distribution is reduced over the pericentromeric regions in Arabidopsis (Yelina et al., 2015) and rice (Si et al., 2015) and that changes in environmental conditions influence recombination (Si et al., 2015).

Sequencing the four meiotic products derived from a meiocyte provides additional information on meiotic recombination because the sequence of the four chromatids that were present in meiosis is obtained. This approach was used in Arabidopsis, maize and budding yeast and revealed the presence of complex template switches and GCs (Mancera et al., 2008; Lu et al., 2012; Li et al., 2015). Finally, recombination rate can be measured at fine-scale (<1 kb) using allele specific PCR amplification from F1 hybrid pollen DNA. This approach confirmed the presence of CO hotspots in Arabidopsis and facilitated the study of specific loci (Choi et al., 2016).

### Meiotic Transcriptome

Microarray and, later, primarily RNA-seq approaches were used to dissect the male meiotic transcriptome based on flower buds, anthers or even isolated meiocytes in various plants while female meiotic transcriptomes were dissected in Arabidopsis and rice (Dukowic-Schulze and Chen, 2014). All studies revealed a complex picture of the meiotic transcriptome, i.e., a large number of genes are expressed and hundreds to thousands of transcripts are differentially expressed. The picture is even more complex, as a high number of mitochondria-encoded genes possibly constituting a source of energy for meiotic progression (Dukowic-Schulze et al., 2014), transposable elements (Chen et al., 2010; Yang et al., 2011), and (long) non-coding RNAs (Dukowic-Schulze et al., 2016; Flórez-Zapata et al., 2016; Wu et al., 2017) are differentially expressed. Changes in chromatin and chromosome organization may cause a general chromatin de-repression accounting for this complexity including an elevated expression of transposable elements in meiocytes (Chen et al., 2010; Yang et al., 2011; Dukowic-Schulze et al., 2014).

RNA interference (RNAi) machinery components and miRNAs are differentially expressed during meiosis (Dukowic-Schulze et al., 2014, 2016; Flórez-Zapata et al., 2016; Wu et al., 2017) and mutations in RNAi machinery components result in aberrant meiotic progression, chromatin structure or HR (Nonomura et al., 2007; Singh et al., 2011; Oliver et al., 2014, 2017), indicating that the RNAi machinery plays a role in meiosis. In male monocot meiotic transcriptomes phasiRNAs are detected that originate from a few hundred dispersed intergenic, non-repetitive regions (phasiRNA loci) and apparently do not target any genes but instead mediate in *cis* DNA methylation at their loci of origin (Dukowic-Schulze et al., 2016). Various long non-coding RNAs were differentially expressed in meiocytes of three sunflower genotypes differing in meiotic recombination rates (Flórez-Zapata et al., 2016), suggesting a link between long non-coding RNAs and meiosis. What specific roles non-coding RNAs play needs to be elucidated. To decipher their localization and dynamics, single molecule FISH, so far limited to root cells, may be used to visualize and quantify RNA molecules at the single-cell level (Duncan et al., 2016). Down-regulation and/or over-expression of candidate loci may help to dissect their function during meiosis.

To identify key meiotic genes, meiotic transcriptomes from different genetic backgrounds (e.g., mutant vs. wildtype, diploid vs. polyploid, treated vs. untreated) were compared. Comparative meiotic transcriptomics between synthetic tetraploid *B. rapa* with aberrant meiosis and its fertile diploid progenitors identified more than 4500 differentially expressed genes including eleven known meiotic genes (Braynen et al., 2017). ZYP1 and SYN1 expressions were upregulated both of which were also implicated as potential candidates for preventing polyploidy-related chromosome segregation challenges in Arabidopsis (Yant et al., 2013). Maize and rice *am1* meiotic transcriptomes were compared to their respective wild-type (Nan et al., 2011; Zhang et al., 2015). In rice HEI10, MSH5, ZIP4, and PSS1 while in maize SMC3, ATR, ATM, RMI1, and MPA1 were identified among thousands of differentially expressed genes as meiotic candidates, suggesting that AM1 plays a role in modulating the expression of many critical meiotic genes in a species-specific dependent manner. Rice ovule transcriptomes from different wild-type genotypes and various female-sterile lines revealed a high number of differentially expressed genes and miRNAs (Yang et al., 2016, 2017; Zhu et al., 2017). Even by performing comparative meiotic transcriptomics in various plants, the complexity of meiotic transcriptomes is astonishing. Thus, whether all identified genes in meiotic transcriptomes are indeed essential for meiosis or whether the large number of detected transcripts is the result of global de-repression of chromatin during meiosis needs to be elucidated.

Reducing sample complexity could be achieved through single cell-type isolation. Potentially flow-cytometric isolation of meiocytes based on plants expressing meiotic proteins tagged with FPs (**Figure 2**), meiotic protein immunolabelling of meiocytes in solution pre-sorting, or the INTACT method (Deal and Henikoff, 2010) could allow enrichment for distinct meiocyte fractions. However, even isolated meiocytes represent a pool of different cells at various meiotic stages or at least sub stages and so far no studies have reported isolation of meiotic cells or nuclei so it is unclear whether these techniques could be applied to meiocytes.

## Engineering Plants

### Generating Mutants

Reverse genetic approaches are key to identifying genes associated with a phenotype. They are widely used in Arabidopsis and reverse genetic resources, including several targeted induced local lesions in the genome (TILLING) populations, were developed in various crops in recent years (Jacob et al., 2018). The higher level of ploidy and higher gene copy numbers makes polyploid plants such as wheat more tolerant to a high density of mutations than diploid plants such as barley and it reduces the proportion of infertile or embryonic lethal M2 plants. However, the complexity of polyploid genomes renders the detection of the mutation sites challenging. Development of exome capture, that scans the exons to identify mutations disrupting coding regions, has facilitated e.g., the identification of EMS-mediated mutation sites in tetraploid and hexaploid TILLING wheat populations (Krasileva et al., 2017).

However, these mutant populations present several limitations. First, the mutant lines have a high density of mutations and several rounds of backcrosses with a non-mutant line are required before full characterisation of a phenotype. In addition, TILLING approaches have limitations in targeting several copies of a gene of interest and crosses of independent mutant lines are then required to combine mutations. As an alternative, RNAi-based gene silencing and Clustered Regularly Interspaced Short Palindromic Repeats (CRISPR)/Cas9 system are efficient to knockdown or knockout several genes simultaneously. These techniques are promising mutagenesis tools for polyploid species such as wheat and have been used to study meiotic genes e.g., in barley (Fu et al., 2007; Barakate et al., 2014; Wang et al., 2014; Lawrenson et al., 2015). Unfortunately, these techniques typically rely on stable plant transformation and only a few institutions have the technology to transform e.g., cereals with exogenous DNAs.

Several mutations in the meiotic class II CO pathway (Seguela-Arnaud et al., 2015; Fernandes et al., 2017) and overexpression of the E3 ligase HEI10 (Ziolkowski et al., 2017), involved in formation of class I COs, cause an increase in genome-wide CO rate in Arabidopsis. Both class I and II CO pathways are attractive targets to manipulate recombination in crops (Serra et al., 2018). However, several components of the class II CO pathway are also involved in somatic DNA repair and maintenance of genome stability. Thus, targeted delivery systems to modulate activity of specific genes in reproductive tissues are needed. Virus-induced gene silencing (VIGS) has emerged as a rapid and inexpensive transient gene knock-down system in plants by exploiting plant defense mechanism based on RNAi against virus infection (Lee et al., 2012). Barley stripe mosaic virus was successfully engineered to manipulate meiotic genes in the wheat cultivar ‘Chinese Spring’ and other wheat genotypes (Bennypaul et al., 2012; Bhullar et al., 2014). Additionally, VIGS can transiently knockdown essential genes during development, especially important if the genes to be silenced are involved in epigenetic marks (e.g., DNA methylation) or genome stability, as a stable knockout could otherwise lead to (embryonic) lethality or loss of fertility.

### Targeted Recombination

Conventional methods to introduce genetic diversity into plant genomes are laborious and depend on the rate of meiotic recombination. In wheat meiotic recombination is repressed over heterochromatin regions preventing the introduction of genetic diversity into genes present within these regions (Choulet et al., 2014). Therefore, new techniques to engineer the genome and manipulate recombination landscapes are needed. Recent genomic data suggest that DSB rate over genes positively correlates with CO rate in maize (He et al., 2017). It is possible that by influencing the rate of DSBs this will in turn cause a change in COs over targeted regions.

In budding yeast, the formation of artificial DSBs using site specific endonucleases is sufficient to create meiotic recombination in cold spot regions (Sarno et al., 2017). The recombination frequency was variable between employed endonucleases and targeted chromosomal regions suggesting that local factors may influence the conversion rate of DSBs to COs. In plants three classes of site-specific endonucleases, Zinc finger nucleases, transcription activator-like effector nucleases (TALENs) and CRISPR/Cas9 are used to edit the genome of plant species such as Arabidopsis, rice and maize (Sun et al., 2016). These nucleases generate DSBs in the targeted nucleotide sequences and the DSBs are either repaired in an error-prone repair pathway or are repaired and edited by HR using a transgenic donor as DNA repair template (Fauser et al., 2012; Sun et al., 2016). In meiosis, DSBs are preferentially repaired by HR and any additional DSB formed can potentially become a CO. The proven efficiency of these site-specific nucleases to generate somatic DSBs suggests that they may also be used during meiosis to manipulate CO rate. To increase the conversion of artificial DSBs to COs, additional local factors may need to be modified. For example, local nucleosome occupancy can be altered with chromatin remodelers. Pro-CO factors like HEI10 could be site-specific targeted alongside SPO11 to promote both DSB formation and maturation of IH recombination molecules to COs, or artificial DSBs could be triggered in hyper-recombination plants (e.g., *fancm*, *recq4*, and/or *figl1*) increasing the likelihood of a DSB maturing into a CO. Although the potential to target recombination toward genes in crops is significant as it can reduce the cost and time to produce novel plant varieties, several challenges exist and the application of these techniques to plant meiosis remains to be demonstrated.

### Making Use of Natural Variation

*Arabidopsis thaliana* is found in many different natural habitats showing extensive intraspecific variation in measurable traits that differ quantitatively between accessions (Weigel, 2012; The 1001 Genomes Consortium, 2016). The genomes of a total of 1,135 natural inbred *A. thaliana* lines from Eurasia, North Africa and colonized North America and 3010 accessions of Asian cultivated rice were sequenced (The 1001 Genomes Consortium, 2016; Wang et al., 2018). Comparative genomic analysis revealed a high degree of intraspecific genetic divergence with the presence of SNPs, small and large insertions/deletions, copy number variations and structural variations. Interestingly a large number of genes contain SNPs which introduce premature stop codons predicted to form non-functional proteins, SNPs which alter translational start sites or donor/acceptor splicing sites predicted to form alternative transcripts (Cao et al., 2011). Phenotypic differences in Arabidopsis have also been associated with variation in epigenetic marks at specific loci, so-called epialleles (O’Malley and Ecker, 2012; Weigel and Colot, 2012; Dubin et al., 2015). A direct relationship between the phenotypic trait and the extent of DNA methylation was demonstrated for most but not all epialleles suggesting that other epigenetic factors are probably also involved (O’Malley and Ecker, 2012; Weigel and Colot, 2012).

Intraspecific variation in meiotic recombination frequency was found in various plants (Lawrence et al., 2017). For instance, F1 Arabidopsis hybrids from 32 diverse accessions revealed extensive variation in CO rate (Ziolkowski et al., 2015). Several confounding factors could account for these CO changes. For example, each ecotype has distinct genetic information and the degree of polymorphism represses CO rate (Lawrence et al., 2017). F1 hybrid plants arising from parents with potentially distinct epigenomes could also influence recombination locally (Cortijo et al., 2014; Yelina et al., 2015). In addition, trans-acting factors exerted by polymorphic loci can modulate recombination. The first plant quantitative trait loci for recombination was recently identified as HEI10 and over-expression of HEI10 in Arabidopsis causes a greater than twofold increase in CO formation genome-wide (Ziolkowski et al., 2017). Additional trans-acting factors probably exist. For example, MSH2 presents gene copy number variations among Arabidopsis accessions and represses recombination between divergent genomes (Emmanuel et al., 2006; Zmienko et al., 2016).

## Novel Approaches Exploiting Non-Model Plants

B chromosomes (supernumerary chromosomes) found across a variety of animals, plants and fungi do not recombine with the standard “A” chromosomes (Jones et al., 2008). Numerous reports in plants suggest an impact of B’s on meiotic recombination behavior (chiasma frequency and/or distribution) of homologous and homeologous A chromosomes in diploid, polyploid and inter-species hybrids (Jones and Rees, 1982; Jones et al., 2008). Genetic and genotypic A–B interactions seem to impact chiasma number and distribution (Ortiz et al., 1996; Kousaka and Endo, 2012). B chromosomes in rye are transcriptionally active containing several B-enriched transcriptionally active tandem repeats (Martis et al., 2012; Klemme et al., 2013), transcribed transposable elements (Ma et al., 2017), and long non-coding RNAs (Carchilan et al., 2007) all of which are predominantly found in anthers. B-encoded pseudogene-like fragments and genes are transcribed in a tissue-type and genotype-specific manner and can cause down-/upregulation of A-located counterparts (Banaei-Moghaddam et al., 2013; Ma et al., 2017). More than 300 B-encoded anther transcripts show similarity to proteins with functional annotation. Among them are SHOC1, PCH2, or SCC3 known to be involved in meiosis and further candidates relating to DNA methylation, chromatin remodeling, the UPS or DNA repair (Ma et al., 2017). Since the effect of B’s on the host recombination landscape seems to have a genetic basis and is often dosage dependent, together with B’s encoding non-coding RNAs and various genes including known meiotic genes, it seems likely that B’s may have a direct impact on the recombination machinery of its host. Further studies could shed light on meiotic recombination mechanisms in the presence of B’s. Despite the potential of B’s as tools for manipulating meiotic recombination in breeding processes, there has been limited utilization of this knowledge in crop breeding (Jones et al., 2008). By standard crossing schemes they could be easily introduced and removed without recombining with As.

Although HR is conserved across species (Mercier et al., 2015), differences in progression of meiosis and recombination intermediates are found between plant species (Lambing et al., 2017). In Arabidopsis *figl1* shows an increase in meiotic recombination without affecting fertility (Girard et al., 2015), whereas in rice *figl1* male meiotic chromosomes undergo fragmentation causing male infertility (Zhang P. et al., 2017). In Arabidopsis and barley reduced ZYP1 levels result in reduced CO numbers (Higgins et al., 2005; Barakate et al., 2014), whereas in rice ZEP1 depletion leads to an increase in CO numbers (Wang et al., 2010). Meiotic studies in non-model plant species also revealed differences e.g., in centromere/kinetochore regulation during meiotic divisions leading to altered chromosome segregation patterns (Cabral et al., 2014; Heckmann et al., 2014; Cuacos et al., 2015; Marques and Pedrosa-Harand, 2016). In the European larch, as in most gymnosperms, female meiosis starts and completes during spring whereas male meiosis starts in autumn and finishes in spring and is characterized by a “diffuse stage” during diplotene lasting ~5 months (Zhang et al., 2008; Kołowerzo-Lubnau et al., 2015). This long male diplotene stage is characterized by microsporocyte growth, synthesis and accumulation of mRNAs and proteins, and changes in chromatin conformation, i.e., condensation cycles of contraction and relaxation correlating with transcriptional activity. Further studies in the European larch or other gymnosperms may reveal additional insights into chromatin dynamics and transcription during meiosis and differences in induction and progression of male vs. female meiosis. Due to slow-paced progression during prophase I, for instance, assembly/disassembly of the bouquet and the SC or formation and dissolution of interlocks could be studied in detail. In numerous plant species, primarily during male prophase I, cytomixis occurs, i.e., migration of whole nuclei, chromosomes and/or chromatin between plant cells through intercellular channels (cytomictic channels) resulting in the formation of unreduced, polyploid, aneuploid or sterile pollen (Mursalimov et al., 2015; Mursalimov and Deineko, 2017). How cytomixis is regulated or interconnected to meiotic progression is unclear. In translocation heterozygote plants CO formation is restricted to distinct chromosome regions commonly leading to long chromosome chains (Stack and Soulliere, 1984; Rauwolf et al., 2011; Golczyk et al., 2014). In Oenothera meiosis, for instance, a spatiotemporal genome compartmentation occurs, i.e., chromosomes are organized in two epigenetically distinct regions, uneven chromosome condensation occurs and COs occur at end-segments of chromosomes roughly at the junction between the two chromatin fractions, resulting in chromosome chains/rings (Rauwolf et al., 2011; Golczyk et al., 2014). How this tightly restricted CO localization is achieved or how balanced chromosome segregation occurs is unclear. Moreover, in closely related species such as Allium differences in recombination patterns are found, i.e., either proximal or interstitial/distal CO (Albini and Jones, 1987), possibly offering models to get a better understand CO patterning control in closely related species.

Thus, although general mechanisms of meiosis and HR are conserved, studies in different species, including non-model species, may widen our knowledge of plant meiosis revealing differences and similarities and possibly enabling a deeper understanding of underlying mechanisms.

## Concluding Remarks and Future Perspectives

In recent years studies, mainly in Arabidopsis but also in selected crops and non-model species, have increased our understanding of plant meiotic progression and recombination and many genes and factors involved in these processes were identified. However, much remains to be learned, even though our current knowledge may provide a basic foundation to explore whether meiotic recombination in crops can be manipulated to improve and accelerate plant breeding programs. Differences in plant genome organization (particularly repetitive DNA content and ploidy level) accompanied by differences in chromatin and epigenetic features likely account for differences in meiotic progression and recombination patterns. Thus, available and new approaches are needed to investigate the underlying mechanisms and factors responsible for differences and similarities in meiotic progression and recombination between model, crop and non-model plants to ultimately translate our knowledge into crop breeding programs.

## Author Contributions

All authors listed have made a substantial, direct and intellectual contribution to the work, and approved it for publication.

## Conflict of Interest Statement

The authors declare that the research was conducted in the absence of any commercial or financial relationships that could be construed as a potential conflict of interest. The reviewer AH declared a shared affiliation, though no other collaboration, with one of the authors SH to the handling Editor.
